# The complete mitochondrial genome of rare and Critically Endangered *Anilany helenae (Microhylidae)* of Madagascar

**DOI:** 10.1080/23802359.2021.2008841

**Published:** 2022-01-05

**Authors:** Katherine E. Mullin, Daniel Firmin, Nina F. D. White, Frank Hailer, Pablo Orozco-terWengel

**Affiliations:** Cardiff University, School of Biosciences, Sir Martin Evans Building, Museum Avenue, Cardiff, UK

**Keywords:** Mitogenome, amphibian, biodiversity, Madagascar, taxonomy

## Abstract

*Anilany helenae* is a Critically Endangered frog native to the central highlands of Madagascar. Due to ongoing habitat loss of its known range, this species’ population is considered declining, while little is known about its ecology, behavior, and taxonomy. Within the context of developing tools that can aid the conservation of Madagascar’s amphibian fauna, and add to the continued understanding of their taxonomy, we assembled its complete mitochondrial genome (Genbank Accession number MZ751042). This contributes the first complete mitochondrial genome of a microhylid from Madagascar, despite there being over 100 species in the Cophylinae subfamily alone. *Anilany helenae*’s circular mitochondrial genome is 17,519 bp long, contains 37 genes, and exhibits differences in gene arrangement compared with other microhylids, including the placement of protein coding genes nad1 and nad2. A phylogeny of the 13 protein coding genes of the few Madagascan anuran mitogenomes available, along with species from Africa and East Asia, places *A. helenae* along with the New Guinean *Mantophryne lateralis* in a basal position with respect to the other microhylids in the tree.

*Anilany helenae* Vallan 2000 is a Critically Endangered species listed on the Zoological Society of London’s Evolutionarily Distinct and Globally Endangered (EDGE) of Existence programme (IUCN 2016; EDGE 2020). It is a small microhylid frog (SVL 10–14 mm) in the Cophylinae subfamily, endemic to Madagascar. The species is found in the island’s central highlands, where it is thought to be micro-endemic to the Ambohitantely Special Reserve area. Little is known about this species, its ecology, range, or taxonomy. Its IUCN Red List assessment describes it as being known from just two small habitat fragments in the vicinity of Ambohitantely at 1,500 m asl (IUCN 2016; Andreone et al. 2008), however recently *A. helenae* was found in multiple forest fragments within Ambohitantely (K Mullin, unpublished) as well as 10 km away in Ankafobe, in a small threatened forest fragment (Mullin et al. 2021). Due to its limited range and the vulnerability of the small habitat fragments it is found in (due to ranching, forest exploitation, fires, and fire suppression), its population is considered declining (IUCN 2016). A recent acoustic study estimated its population size to be just 388 males in the main and largest fragment at Ambohitantely (Barata et al. in press).

This species was originally described as being part of the *Stumpffia* genus, but Scherz et al. (2016) proposed its placement in its own genus, *Anilany.* It is thought that there are further species among this genus, but these are yet to be formally described (Pers. Comm. Scherz). Due to frequent discovery and description of novel species, the taxonomy of Madagascar’s Cophylinae subfamily is continuously debated and revised. Here we describe the complete mitogenome of *A. helenae*, contributing to the continued research into Madagascar’s anuran taxonomy.

A specimen (KAMU21) was collected by Katherine Mullin from the main forest block in Ambohitantely on 13 April 2019 at −18.19694, 47.28413 during visual encounter surveys. The specimen was found in the leaf litter on the forest floor. This specimen and the DNA has been deposited in the archives of the Molecular Ecology Lab, Cardiff University. Genomic DNA was extracted from a tissue sample using the QIAGEN DNeasy Blood & Tissue Kit protocol. Whole genome paired-end Illumina sequencing (150 PE) was performed by Novogene, Hong Kong. The raw reads were assembled using the *de novo* NOVOPlasty organelle assembler and heteroplasmy caller (Dierckxsens et al. 2016). Given no reference mitogenome genome was available for this species, or a close relative, a previously generated Cytochrome c oxidase1 (COX1) partial gene sequence was used as an input seed for NOVOPlasty. The output from NOVOplasty was circularized reaching a total contig length of 17,519 bp using 25,730 reads, achieving a mean coverage of 687.7 (Std. Dev. 254.1).

The mitogenome had a GC content of 41.1%, with individual base percentages of: G − 12.9%, C − 28.2%, A − 30.3%, T − 28.5%. The MITOS WebServer (Bernt et al. 2013) was used to guide the annotation of a total of 37 genes. Thirteen protein coding genes, two rRNA genes, and 22 tRNA genes were identified.

The order and orientation of genes show variation when compared with the anurans in our phylogenetic tree (Figure 1). Similar mitochondrial gene order rearrangements have been described for other Madagascan amphibians, such as members of the genus *Mantella* (Kurabayashi et al. 2006). *Anilany helenae’s* mitogenome shows a different gene order than other microhylids, spanning the region from Cytochrome b (*cob)* to trnN(aac), which is consistent with the region of genomic reorganization known for seven mantellid genera from Madagascar (Kurabayashi et al. 2008). In *A. helenae* the protein coding genes NADH dehydrogenase subunit 1 (nad1), NADH dehydrogenase subunit 2 (nad2) and the 16S rRNA genes show rearrangement, with nad2 placed after cob, and before (rather than after) 16S and nad1. Further sequencing with longer reads may be required to fully resolve the gene order and any duplications.

**Figure 1.**
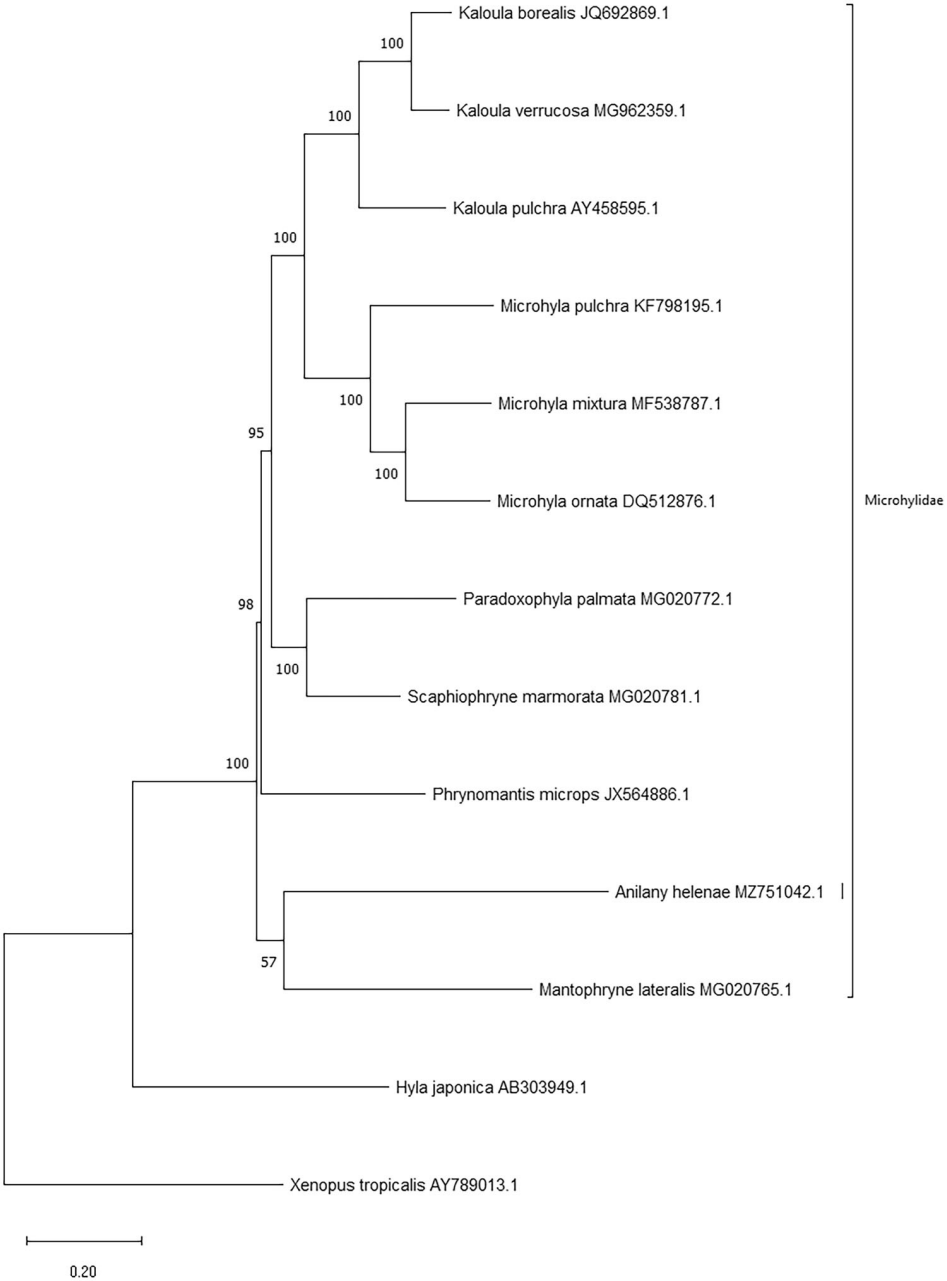
Maximum likelihood phylogeny using a 10,934 bp alignment of mitogenome protein coding genes. The outgroup is distant relative *Xenopus tropicalis* (AY789013.1).

A total of 11 microhylidae (7 from East Asia, 1 from mainland Africa and 2 from Madagascar, in addition to *A. helenae*) and 1 hylidae species were used to infer phylogenetic relationships (Figure 1). The 13 protein coding gene sequences were taken from Genbank, concatenated by hand, aligned using the ClustalW algorithm and trimmed in MEGAX (Kumar et al. 2018), to a total of 11,373 positions. MEGAX was used to construct a maximum likelihood phylogeny with complete gap deletion (10,934 positions remaining), using the GTR + G + I model of DNA sequence evolution, and carrying out 100 bootstrap replicates to determine statistical branch support. *Anilany helenae* formed a clade with *Mantophryne lateralis* which is endemic to New Guinea, however we note that the support is low (57 %), and that this phylogeny should be reexamined with increased taxon sampling, including additional Madagascar Cophylinae species such as close relatives in the *Stumpffia* and *Anodonthyla* genera. Our results support the ongoing taxonomic work on Madagascar’s amphibian fauna.

## Data Availability

Mitogenome data supporting this study are openly available in GenBank of NCBI at https://www.ncbi.nlm.nih.gov/nuccore/MZ751042 under the accession number MZ751042. The associated BioProject, SRA, and BioSample numbers are PRJNA741236, SRR14908290, and SAMN19862015, respectively.

## Geolocation information

This species is a micro-endemic of the Ambohitantely Special Reserve region on the central plateau of Madagascar.
